# Tetra­kis[μ_2_-5-nitro-2-(phenyl­sulfan­yl)benzoato-κ^2^ *O*:*O*′]bis­[(aceto­nitrile-κ*N*)copper(II)]

**DOI:** 10.1107/S2414314620008019

**Published:** 2020-06-23

**Authors:** Danrui Xu, Jun Gao, Sihui Long

**Affiliations:** a School of Chemical Engineering and Pharmacy, Wuhan Institute of Technology, Wuhan, Hubei 430205, People’s Republic of China; Vienna University of Technology, Austria

**Keywords:** crystal structure, copper, bis-monodentate coordination, dimeric complex, C—H⋯O hydogen bonding

## Abstract

The title compound, [Cu_2_(μ_2_-O_2_CC_12_H_8_NO_2_S)_4_(CH_3_CN)_2_], consists of a dinuclear and centrosymmetric complex based on two Cu^II^ atoms coordinated by four 5-nitro-2-phenyl­sulfanyl-benzoate anions and two aceto­nitrile ligands. Each benzoate anion acts as a bis-monodentate ligand while each aceto­nitrile acts as a monodentate ligand, leading to a square-pyramidal NO_4_ coordination environment for each Cu^II^ atom with the aceto­nitrile N atom at the apex. The intra­molecular Cu⋯Cu distance in the dimer is 2.6478 (3) Å. The cohesion of the crystal structure is ensured by _(phen­yl)_C—H⋯O_(nitro)_ hydrogen bonds and _(phen­yl)_C—H⋯π inter­actions.

## Abstract 2

Pairs of bis-monodentate 5-nitro-2-phenyl­sulfanyl-benzoate ligands bridge two Cu^II^ atoms related by inversion symmetry. The square-pyramidal coordination geometry of each copper(II) atom is completed by an acetronitile ligand at the apex. The intra­molecular Cu⋯Cu distance in the dimer is 2.6478 (3) Å.

## Graphical abstract

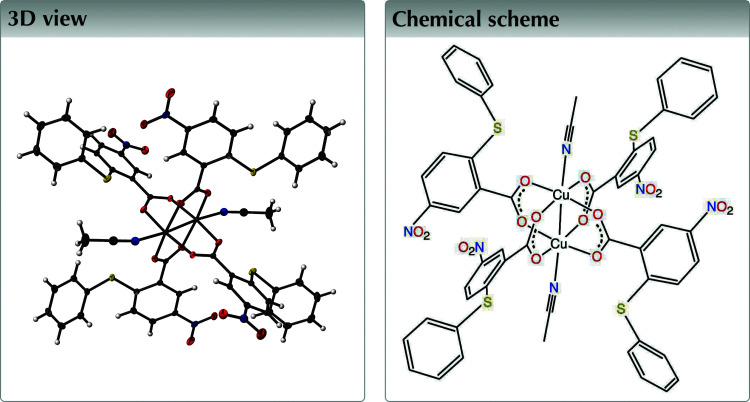

## Structure description

The title compound was obtained serendipitously during efforts to make 5-nitro-2-phenyl­sulfanyl-benzoic acid by reacting 2-bromo-5-nitro-benzoic acid with benzene­thiol through a modified Ullmann reaction with Cu/Cu_2_O as catalyst, K_2_CO_3_ as base, and eth­oxy­ethanol as solvent (Liu *et al.*, 2007 ▸). The complex likely formed between Cu^II^ generated *in situ* and 5-nitro-2-phenyl­sulfanyl-benzoate.

**Figure 1:**
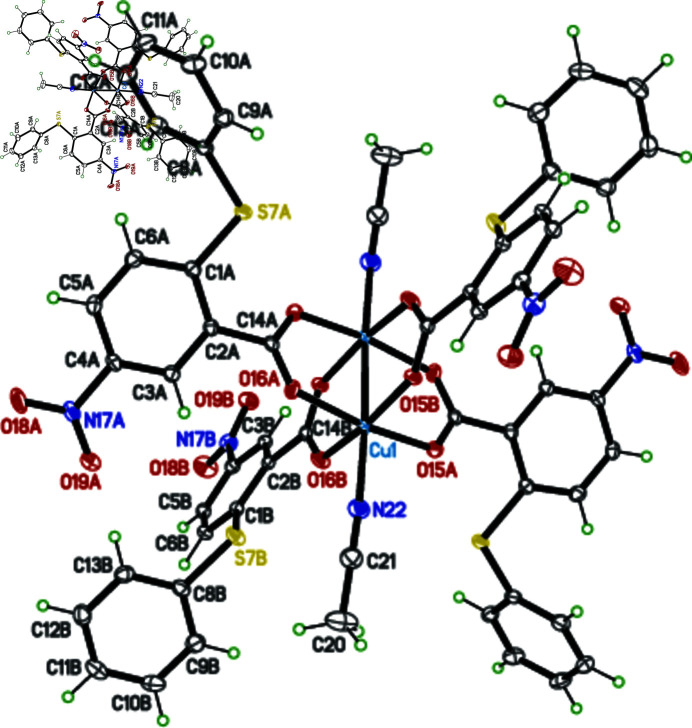
The mol­ecular structure of the binuclear title complex, with displacement ellipsoids drawn at the 50% probability level (arbitrary spheres for the H atoms). Non-labelled atoms are generated by inversion symmetry (symmetry code: −*x* + 1, −*y* + 2, −*z*).

**Figure 2:**
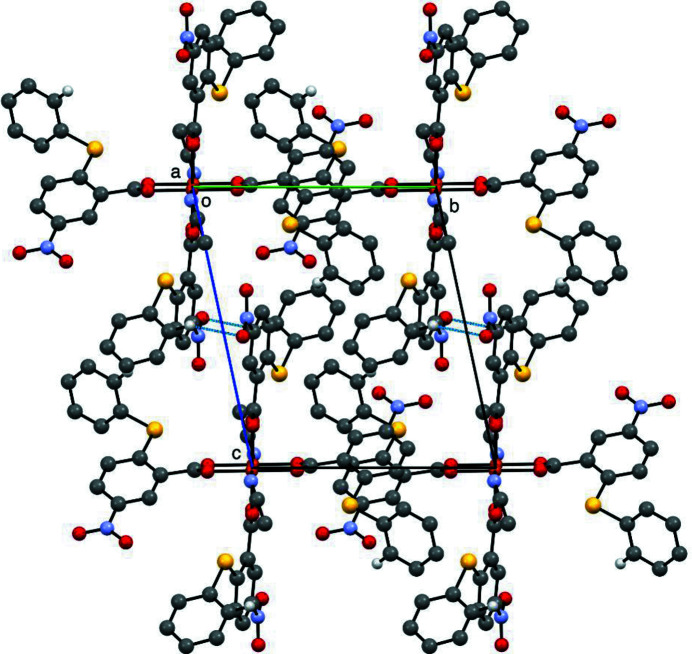
Packing of the mol­ecules in the title compound, with inter­molecular C—H⋯O hydrogen bonds indicated by blue dashed lines (H atoms not participating in hydrogen bonding are omitted).

The title compound is a centrosymmetric dinuclear Cu^II^ complex with two pairs of bis-monodentate 5-nitro-2-phenyl­sulfanyl-benzoate anions and a pair of aceto­nitrile mol­ecules as ligands (Fig. 1 ▸). The coordination of each copper(II) atom is square-pyramidal, with the aceto­nitrile N atom at the apex of the NO_4_ coordination set. The intra­molecular Cu⋯Cu distance in the dimer is 2.6478 (3) Å (Fig. 1 ▸). The aromatic rings of the two independent benzoate moieties (denoted by suffix *A* and *B* for the two anions in the asymmetric unit) are nearly perpendicular with a dihedral angle of 86.55 (5)°. The negative charge of the carboxyl­ate group is delocalized over the two O atoms in each of the anions, as indicated by the nearly identical length of the two C—O bonds [1.2574 (17) and 1.2624 (17) Å for mol­ecule *A* and 1.2574 (17) and 1.2655 (16) Å for mol­ecule *B*]. A weak inter­molecular hydrogen bond is formed between the C9*B*—H9*B* group of a phenyl ring and the O19*B* atom of an inversion-related (−*x* + 1, −*y* + 2, −*z* + 1) NO_2_ group (Table 1 ▸), leading to the formation of supra­molecular pillars along [100] and [010] (Fig. 2 ▸). An additional C—H⋯π inter­action between C10*A*—H10*A* and the centroid of a neighbouring phenyl ring (Table 1 ▸) consolidates the three-dimensional network structure.

The mol­ecular structure of the title complex is similar to that of tetra­kis­(μ_2_-benzoato-*O,O*’)-bis­(di­methyl­sulfoxide)­dicopper(II) (Reyes-Ortega *et al.*, 2005 ▸) and other related copper complexes (Vives *et al.*, 2003 ▸).

## Synthesis and crystallization

**Figure 3:**
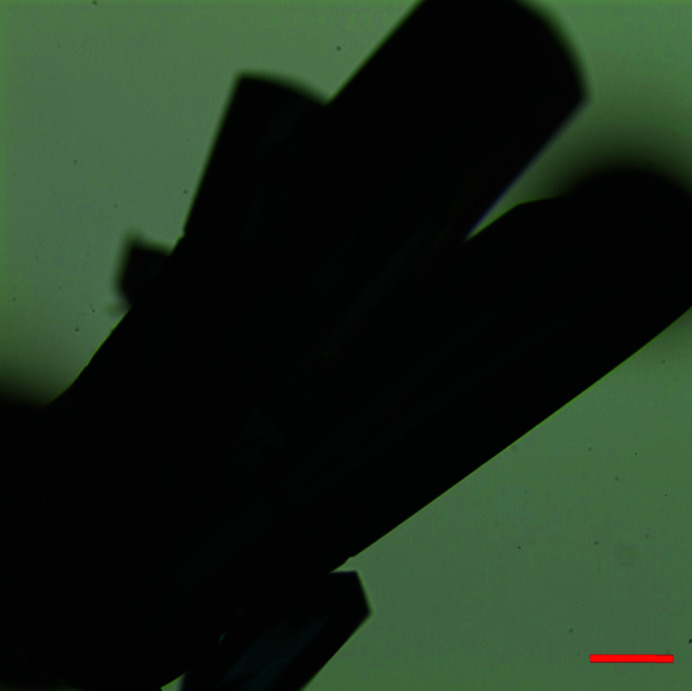
Crystals of the title complex. The length of the red scale bar represents 0.1 mm.

A mixture of benzene­thiol (1.25 g, 11.4 mmol), 2-bromo-5-nitro-benzoic acid (2.16 g, 8.8 mmol), K_2_CO_3_ (1.21 g, 8.8 mmol), Cu powder (51 mg, 0.8 mmol), Cu_2_O (38 mg, 0.4 mmol) and 2-eth­oxy­ethanol (3 ml) was heated to 403 K for 24 h. The reaction was cooled down to room temperature, and the solvent was removed under reduced pressure. The residue was poured into water (30 ml), treated with charcoal, and the resulting suspension was filtered through Celite. Acidification of the filtrate with dilute HCl (pH 5) gave a bluish precipitate (crude product). The crude product was dissolved in aqueous Na_2_CO_3_ solution (5%_wt_, 100 ml) and the solution was filtered through Celite and subjected to acidification and subsequent precipitation to give a pure product. Blue rod-shaped crystals were grown from CH_3_CN solution by slow evaporation (Fig. 3 ▸).

## Refinement

Crystal data, data collection and structure refinement details are summarized in Table 2 ▸.

## Supplementary Material

Crystal structure: contains datablock(s) global, I. DOI: 10.1107/S2414314620008019/wm4131sup1.cif

Structure factors: contains datablock(s) I. DOI: 10.1107/S2414314620008019/wm4131Isup2.hkl

CCDC reference: 2009944

Additional supporting information:  crystallographic information; 3D view; checkCIF report

## Figures and Tables

**Table 1:** Hydrogen-bond geometry (Å, °) *Cg*4 is the centroid of the C8*B*–C13*B* ring.

*D*—H⋯*A*	*D*—H	H⋯*A*	*D*⋯*A*	*D*—H⋯*A*
C9*B*—H9*B*⋯O19*B* ^i^	0.95	2.39	3.2351 (19)	148
C10—H10*A*⋯*Cg*4^ii^	0.95	2.63	3.4171 (17)	140

Symmetry codes: (i) 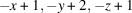 ; (ii) 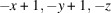 .

**Table 2:** Experimental details

Crystal data	
Chemical formula	[Cu_2_(C_13_H_8_NO_4_S)_4_(C_2_H_3_N)_2_]
*M*_r_	1306.24
Crystal system, space group	Triclinic, *P* 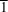
Temperature (K)	90
*a*, *b*, *c* (Å)	10.7377 (1), 11.0957 (1), 12.4857 (2)
α, β, γ (°)	77.9948 (5), 88.1362 (5), 72.3710 (5)
*V* (Å^3^)	1385.97 (3)
*Z*	1
Radiation type	Mo *K*α
μ (mm^−1^)	1.00
Crystal size (mm)	0.50 × 0.40 × 0.20

Data collection	
Diffractometer	Nonius KappaCCD diffractometer
Absorption correction	Multi-scan (*SCALEPACK*; Otwinowski & Minor, 1997 ▸)
*T*_min_, *T*_max_	0.636, 0.826
No. of measured, independent and observed [*I* > 2σ(*I*)] reflections	12571, 6335, 5914
*R*_int_	0.016
(sin θ/λ)_max_ (Å^−1^)	0.650

Refinement	
*R*[*F* ^2^ > 2σ(*F* ^2^)], *wR*(*F* ^2^), *S*	0.023, 0.061, 1.05
No. of reflections	6335
No. of parameters	380
H-atom treatment	H-atom parameters constrained
Δρ_max_, Δρ_min_ (e Å^−3^)	0.36, −0.39

Computer programs: *COLLECT* (Nonius, 2002 ▸), *DENZO-SMN* (Otwinowski & Minor, 1997 ▸), *XP* in *SHELXTL* and *SHELXS97* (Sheldrick, 2008 ▸), *SHELXL2018/3* (Sheldrick, 2015 ▸), *Mercury* (Macrae *et al.*, 2020 ▸) and *publCIF* (Westrip, 2010 ▸).

## full crystallographic data

### Crystal data

**Table:**

[Cu_2_(C_13_H_8_NO_4_S)_4_(C_2_H_3_N)_2_]	*Z* = 1
*M**_r_* = 1306.24	*F*(000) = 666
Triclinic, *P*1	*D*_x_ = 1.565 Mg m^−^^3^
*a* = 10.7377 (1) Å	Mo *K*α radiation, λ = 0.71073 Å
*b* = 11.0957 (1) Å	Cell parameters from 6289 reflections
*c* = 12.4857 (2) Å	θ = 1.0–27.5°
α = 77.9948 (5)°	µ = 1.00 mm^−^^1^
β = 88.1362 (5)°	*T* = 90 K
γ = 72.3710 (5)°	Rod, blue
*V* = 1385.97 (3) Å^3^	0.50 × 0.40 × 0.20 mm

### Data collection

**Table:**

Nonius KappaCCD diffractometer	6335 independent reflections
Radiation source: fine-focus sealed tube	5914 reflections with *I* > 2σ(*I*)
Detector resolution: 18 pixels mm^-1^	*R*_int_ = 0.016
ω scans at fixed χ=55°	θ_max_ = 27.5°, θ_min_ = 1.7°
Absorption correction: multi-scan (*Scalepack*; Otwinowski & Minor, 1997)	*h* = −13→13
*T*_min_ = 0.636, *T*_max_ = 0.826	*k* = −14→14
12571 measured reflections	*l* = −16→16

### Refinement

**Table:**

Refinement on *F*^2^	Primary atom site location: structure-invariant direct methods
Least-squares matrix: full	Secondary atom site location: difference Fourier map
*R*[*F*^2^ > 2σ(*F*^2^)] = 0.023	Hydrogen site location: inferred from neighbouring sites
*wR*(*F*^2^) = 0.061	H-atom parameters constrained
*S* = 1.05	*w* = 1/[σ^2^(*F*_o_^2^) + (0.0208*P*)^2^ + 0.9758*P*] where *P* = (*F*_o_^2^ + 2*F*_c_^2^)/3
6335 reflections	(Δ/σ)_max_ = 0.002
380 parameters	Δρ_max_ = 0.36 e Å^−^^3^
0 restraints	Δρ_min_ = −0.39 e Å^−^^3^

### Special details

**Table:**

Geometry. All esds (except the esd in the dihedral angle between two l.s. planes) are estimated using the full covariance matrix. The cell esds are taken into account individually in the estimation of esds in distances, angles and torsion angles; correlations between esds in cell parameters are only used when they are defined by crystal symmetry. An approximate (isotropic) treatment of cell esds is used for estimating esds involving l.s. planes.

### Fractional atomic coordinates and isotropic or equivalent isotropic displacement parameters (Å^2^)

**Table:**

	*x*	*y*	*z*	*U*_iso_*/*U*_eq_	
Cu1	0.37846 (2)	0.99509 (2)	0.01065 (2)	0.00993 (5)	
C20	0.00546 (18)	1.0018 (2)	0.19864 (15)	0.0383 (4)	
H20A	0.021784	0.918044	0.249462	0.057*	
H20B	−0.082418	1.027638	0.165040	0.057*	
H20C	0.012133	1.066883	0.238691	0.057*	
C21	0.10207 (15)	0.99118 (16)	0.11335 (13)	0.0219 (3)	
N22	0.17853 (12)	0.98250 (13)	0.04760 (11)	0.0210 (3)	
C1A	0.73172 (13)	0.54587 (13)	−0.03417 (11)	0.0129 (3)	
C2A	0.63214 (13)	0.61581 (13)	0.02586 (10)	0.0121 (3)	
C3A	0.57027 (13)	0.55063 (13)	0.10719 (11)	0.0131 (3)	
H3A	0.502908	0.597453	0.147404	0.016*	
C4A	0.60775 (13)	0.41742 (13)	0.12878 (11)	0.0135 (3)	
C5A	0.70116 (14)	0.34601 (13)	0.06829 (11)	0.0154 (3)	
H5A	0.723021	0.254424	0.082499	0.018*	
C6A	0.76180 (14)	0.41077 (14)	−0.01307 (12)	0.0161 (3)	
H6A	0.825160	0.362840	−0.055605	0.019*	
S7A	0.81150 (3)	0.63183 (3)	−0.13466 (3)	0.01537 (8)	
C8A	0.90777 (13)	0.50792 (13)	−0.20027 (11)	0.0141 (3)	
C9A	0.87216 (14)	0.50745 (14)	−0.30608 (12)	0.0164 (3)	
H9A	0.796224	0.570717	−0.341701	0.020*	
C10A	0.94849 (14)	0.41366 (15)	−0.35961 (12)	0.0199 (3)	
H10A	0.925925	0.414027	−0.432642	0.024*	
C11A	1.05753 (15)	0.31967 (15)	−0.30620 (13)	0.0220 (3)	
H11A	1.108205	0.254619	−0.342354	0.026*	
C12A	1.09315 (14)	0.31990 (15)	−0.20054 (13)	0.0211 (3)	
H12A	1.167443	0.254741	−0.164227	0.025*	
C13A	1.01995 (14)	0.41563 (15)	−0.14789 (12)	0.0176 (3)	
H13A	1.046003	0.418341	−0.076659	0.021*	
C14A	0.58662 (13)	0.76073 (13)	0.00758 (10)	0.0119 (2)	
O15A	0.32797 (9)	1.18158 (9)	0.00850 (8)	0.0148 (2)	
O16A	0.46503 (10)	0.81172 (9)	0.01343 (8)	0.0167 (2)	
N17A	0.54354 (12)	0.34971 (12)	0.21587 (9)	0.0167 (2)	
O18A	0.57926 (13)	0.23095 (10)	0.23492 (9)	0.0291 (3)	
O19A	0.45741 (11)	0.41481 (10)	0.26574 (9)	0.0218 (2)	
C1B	0.47177 (13)	0.84097 (13)	0.39310 (11)	0.0132 (3)	
C2B	0.55474 (13)	0.90071 (13)	0.32621 (11)	0.0123 (3)	
C3B	0.66210 (13)	0.91810 (13)	0.37284 (11)	0.0132 (3)	
H3B	0.718486	0.957058	0.328018	0.016*	
C4B	0.68656 (13)	0.87855 (13)	0.48456 (11)	0.0135 (3)	
C5B	0.60457 (14)	0.82457 (13)	0.55299 (11)	0.0146 (3)	
H5B	0.620979	0.800878	0.630000	0.018*	
C6B	0.49851 (13)	0.80600 (13)	0.50678 (11)	0.0146 (3)	
H6B	0.442099	0.768585	0.553019	0.017*	
S7B	0.34082 (3)	0.80704 (4)	0.33483 (3)	0.01856 (8)	
C8B	0.28760 (13)	0.71437 (14)	0.45143 (11)	0.0161 (3)	
C9B	0.18868 (14)	0.77613 (15)	0.51410 (13)	0.0203 (3)	
H9B	0.148411	0.866897	0.493867	0.024*	
C10B	0.14911 (15)	0.70463 (16)	0.60623 (13)	0.0228 (3)	
H10B	0.082094	0.746774	0.649382	0.027*	
C11B	0.20682 (15)	0.57216 (16)	0.63558 (12)	0.0221 (3)	
H11B	0.181478	0.523963	0.700033	0.026*	
C12B	0.30193 (15)	0.50986 (15)	0.57050 (13)	0.0210 (3)	
H12B	0.339840	0.418677	0.589475	0.025*	
C13B	0.34171 (14)	0.58063 (14)	0.47772 (12)	0.0177 (3)	
H13B	0.405530	0.537858	0.432525	0.021*	
C14B	0.53438 (13)	0.94456 (13)	0.20461 (11)	0.0127 (3)	
O15B	0.36927 (9)	1.03736 (9)	−0.14980 (8)	0.01443 (19)	
O16B	0.42258 (10)	0.95862 (10)	0.16679 (8)	0.0179 (2)	
N17B	0.80353 (12)	0.89184 (12)	0.53147 (10)	0.0176 (2)	
O18B	0.81739 (12)	0.86967 (12)	0.63151 (9)	0.0276 (3)	
O19B	0.88348 (10)	0.92404 (11)	0.46750 (9)	0.0218 (2)	

### Atomic displacement parameters (Å^2^)

**Table:**

	*U*^11^	*U*^22^	*U*^33^	*U*^12^	*U*^13^	*U*^23^
Cu1	0.01075 (8)	0.00931 (8)	0.00903 (8)	−0.00330 (6)	0.00151 (6)	−0.00015 (6)
C20	0.0299 (9)	0.0581 (13)	0.0299 (9)	−0.0159 (9)	0.0122 (8)	−0.0135 (9)
C21	0.0170 (7)	0.0264 (8)	0.0228 (8)	−0.0082 (6)	−0.0015 (6)	−0.0034 (6)
N22	0.0148 (6)	0.0245 (7)	0.0233 (6)	−0.0080 (5)	0.0009 (5)	−0.0010 (5)
C1A	0.0140 (6)	0.0137 (6)	0.0112 (6)	−0.0047 (5)	0.0003 (5)	−0.0022 (5)
C2A	0.0143 (6)	0.0115 (6)	0.0104 (6)	−0.0037 (5)	−0.0011 (5)	−0.0018 (5)
C3A	0.0150 (6)	0.0133 (6)	0.0105 (6)	−0.0037 (5)	0.0001 (5)	−0.0025 (5)
C4A	0.0173 (6)	0.0136 (6)	0.0099 (6)	−0.0068 (5)	0.0002 (5)	0.0000 (5)
C5A	0.0183 (7)	0.0106 (6)	0.0163 (6)	−0.0035 (5)	−0.0010 (5)	−0.0019 (5)
C6A	0.0169 (7)	0.0140 (7)	0.0177 (7)	−0.0037 (5)	0.0028 (5)	−0.0055 (5)
S7A	0.01914 (17)	0.01343 (16)	0.01462 (16)	−0.00625 (13)	0.00703 (13)	−0.00429 (12)
C8A	0.0141 (6)	0.0146 (6)	0.0146 (6)	−0.0054 (5)	0.0047 (5)	−0.0040 (5)
C9A	0.0135 (6)	0.0179 (7)	0.0165 (7)	−0.0030 (5)	0.0004 (5)	−0.0036 (5)
C10A	0.0186 (7)	0.0262 (8)	0.0175 (7)	−0.0068 (6)	0.0016 (5)	−0.0103 (6)
C11A	0.0177 (7)	0.0212 (8)	0.0286 (8)	−0.0039 (6)	0.0068 (6)	−0.0122 (6)
C12A	0.0136 (7)	0.0202 (7)	0.0256 (8)	−0.0014 (6)	0.0008 (6)	−0.0015 (6)
C13A	0.0154 (7)	0.0230 (7)	0.0142 (6)	−0.0067 (6)	0.0008 (5)	−0.0022 (5)
C14A	0.0165 (6)	0.0115 (6)	0.0070 (6)	−0.0035 (5)	0.0009 (5)	−0.0012 (5)
O15A	0.0164 (5)	0.0110 (5)	0.0177 (5)	−0.0050 (4)	0.0039 (4)	−0.0039 (4)
O16A	0.0149 (5)	0.0102 (5)	0.0231 (5)	−0.0029 (4)	0.0014 (4)	−0.0009 (4)
N17A	0.0243 (6)	0.0150 (6)	0.0116 (5)	−0.0087 (5)	0.0004 (5)	−0.0004 (4)
O18A	0.0527 (8)	0.0130 (5)	0.0222 (6)	−0.0134 (5)	0.0126 (5)	−0.0016 (4)
O19A	0.0243 (5)	0.0208 (5)	0.0188 (5)	−0.0072 (4)	0.0082 (4)	−0.0015 (4)
C1B	0.0130 (6)	0.0118 (6)	0.0127 (6)	−0.0017 (5)	0.0005 (5)	−0.0013 (5)
C2B	0.0140 (6)	0.0098 (6)	0.0109 (6)	−0.0008 (5)	0.0020 (5)	−0.0017 (5)
C3B	0.0149 (6)	0.0108 (6)	0.0133 (6)	−0.0028 (5)	0.0034 (5)	−0.0031 (5)
C4B	0.0135 (6)	0.0125 (6)	0.0147 (6)	−0.0032 (5)	0.0003 (5)	−0.0043 (5)
C5B	0.0182 (7)	0.0133 (6)	0.0104 (6)	−0.0025 (5)	0.0010 (5)	−0.0016 (5)
C6B	0.0154 (6)	0.0151 (7)	0.0118 (6)	−0.0042 (5)	0.0032 (5)	−0.0007 (5)
S7B	0.01666 (17)	0.02532 (19)	0.01348 (16)	−0.01036 (14)	−0.00195 (13)	0.00278 (13)
C8B	0.0139 (6)	0.0204 (7)	0.0144 (6)	−0.0077 (5)	0.0000 (5)	−0.0006 (5)
C9B	0.0181 (7)	0.0177 (7)	0.0269 (8)	−0.0069 (6)	0.0033 (6)	−0.0066 (6)
C10B	0.0210 (7)	0.0297 (8)	0.0239 (8)	−0.0124 (6)	0.0093 (6)	−0.0134 (6)
C11B	0.0228 (7)	0.0304 (8)	0.0163 (7)	−0.0158 (7)	0.0014 (6)	−0.0007 (6)
C12B	0.0194 (7)	0.0187 (7)	0.0226 (7)	−0.0060 (6)	−0.0029 (6)	0.0015 (6)
C13B	0.0133 (6)	0.0209 (7)	0.0178 (7)	−0.0036 (5)	0.0000 (5)	−0.0041 (6)
C14B	0.0167 (6)	0.0088 (6)	0.0111 (6)	−0.0020 (5)	0.0016 (5)	−0.0018 (5)
O15B	0.0153 (5)	0.0167 (5)	0.0099 (4)	−0.0040 (4)	0.0027 (4)	−0.0015 (4)
O16B	0.0172 (5)	0.0245 (5)	0.0104 (4)	−0.0073 (4)	0.0004 (4)	0.0017 (4)
N17B	0.0187 (6)	0.0177 (6)	0.0180 (6)	−0.0065 (5)	−0.0004 (5)	−0.0059 (5)
O18B	0.0295 (6)	0.0413 (7)	0.0168 (5)	−0.0159 (5)	−0.0035 (4)	−0.0080 (5)
O19B	0.0196 (5)	0.0248 (6)	0.0243 (5)	−0.0116 (4)	0.0030 (4)	−0.0053 (4)

### Geometric parameters (Å, º)

**Table:**

Cu1—O16B	1.9505 (10)	C13A—H13A	0.9500
Cu1—O16A	1.9576 (10)	C14A—O15A^i^	1.2574 (17)
Cu1—O15B	1.9588 (9)	C14A—O16A	1.2624 (17)
Cu1—O15A	1.9685 (10)	N17A—O18A	1.2287 (16)
Cu1—N22	2.2206 (12)	N17A—O19A	1.2287 (16)
Cu1—Cu1^i^	2.6478 (3)	C1B—C6B	1.4062 (18)
C20—C21	1.460 (2)	C1B—C2B	1.4176 (18)
C20—H20A	0.9800	C1B—S7B	1.7746 (14)
C20—H20B	0.9800	C2B—C3B	1.3893 (19)
C20—H20C	0.9800	C2B—C14B	1.4966 (18)
C21—N22	1.139 (2)	C3B—C4B	1.3808 (19)
C1A—C6A	1.4032 (19)	C3B—H3B	0.9500
C1A—C2A	1.4123 (18)	C4B—C5B	1.3862 (19)
C1A—S7A	1.7734 (14)	C4B—N17B	1.4623 (18)
C2A—C3A	1.3919 (18)	C5B—C6B	1.381 (2)
C2A—C14A	1.5017 (18)	C5B—H5B	0.9500
C3A—C4A	1.3785 (19)	C6B—H6B	0.9500
C3A—H3A	0.9500	S7B—C8B	1.7839 (14)
C4A—C5A	1.3865 (19)	C8B—C13B	1.391 (2)
C4A—N17A	1.4654 (17)	C8B—C9B	1.392 (2)
C5A—C6A	1.3816 (19)	C9B—C10B	1.388 (2)
C5A—H5A	0.9500	C9B—H9B	0.9500
C6A—H6A	0.9500	C10B—C11B	1.385 (2)
S7A—C8A	1.7840 (14)	C10B—H10B	0.9500
C8A—C9A	1.3887 (19)	C11B—C12B	1.390 (2)
C8A—C13A	1.397 (2)	C11B—H11B	0.9500
C9A—C10A	1.392 (2)	C12B—C13B	1.391 (2)
C9A—H9A	0.9500	C12B—H12B	0.9500
C10A—C11A	1.387 (2)	C13B—H13B	0.9500
C10A—H10A	0.9500	C14B—O16B	1.2574 (17)
C11A—C12A	1.386 (2)	C14B—O15B^i^	1.2655 (16)
C11A—H11A	0.9500	N17B—O18B	1.2267 (16)
C12A—C13A	1.389 (2)	N17B—O19B	1.2349 (16)
C12A—H12A	0.9500		
			
O16B—Cu1—O16A	87.64 (4)	C12A—C13A—C8A	119.63 (13)
O16B—Cu1—O15B	168.27 (4)	C12A—C13A—H13A	120.2
O16A—Cu1—O15B	91.98 (4)	C8A—C13A—H13A	120.2
O16B—Cu1—O15A	89.65 (4)	O15A^i^—C14A—O16A	126.72 (13)
O16A—Cu1—O15A	168.31 (4)	O15A^i^—C14A—C2A	117.69 (12)
O15B—Cu1—O15A	88.37 (4)	O16A—C14A—C2A	115.55 (12)
O16B—Cu1—N22	90.57 (4)	C14A^i^—O15A—Cu1	119.40 (9)
O16A—Cu1—N22	97.89 (5)	C14A—O16A—Cu1	125.20 (9)
O15B—Cu1—N22	101.10 (4)	O18A—N17A—O19A	123.80 (12)
O15A—Cu1—N22	93.50 (4)	O18A—N17A—C4A	118.00 (12)
O16B—Cu1—Cu1^i^	83.35 (3)	O19A—N17A—C4A	118.21 (11)
O16A—Cu1—Cu1^i^	81.95 (3)	C6B—C1B—C2B	118.05 (12)
O15B—Cu1—Cu1^i^	84.98 (3)	C6B—C1B—S7B	120.91 (10)
O15A—Cu1—Cu1^i^	86.45 (3)	C2B—C1B—S7B	121.02 (10)
N22—Cu1—Cu1^i^	173.92 (4)	C3B—C2B—C1B	119.99 (12)
C21—C20—H20A	109.5	C3B—C2B—C14B	117.13 (12)
C21—C20—H20B	109.5	C1B—C2B—C14B	122.86 (12)
H20A—C20—H20B	109.5	C4B—C3B—C2B	119.77 (12)
C21—C20—H20C	109.5	C4B—C3B—H3B	120.1
H20A—C20—H20C	109.5	C2B—C3B—H3B	120.1
H20B—C20—H20C	109.5	C3B—C4B—C5B	121.79 (13)
N22—C21—C20	179.27 (17)	C3B—C4B—N17B	118.98 (12)
C21—N22—Cu1	142.80 (12)	C5B—C4B—N17B	119.22 (12)
C6A—C1A—C2A	118.39 (12)	C6B—C5B—C4B	118.55 (12)
C6A—C1A—S7A	122.63 (10)	C6B—C5B—H5B	120.7
C2A—C1A—S7A	118.97 (10)	C4B—C5B—H5B	120.7
C3A—C2A—C1A	120.17 (12)	C5B—C6B—C1B	121.77 (13)
C3A—C2A—C14A	116.23 (12)	C5B—C6B—H6B	119.1
C1A—C2A—C14A	123.60 (12)	C1B—C6B—H6B	119.1
C4A—C3A—C2A	119.31 (13)	C1B—S7B—C8B	101.35 (6)
C4A—C3A—H3A	120.3	C13B—C8B—C9B	120.08 (13)
C2A—C3A—H3A	120.3	C13B—C8B—S7B	120.24 (11)
C3A—C4A—C5A	121.98 (12)	C9B—C8B—S7B	119.66 (11)
C3A—C4A—N17A	118.85 (12)	C10B—C9B—C8B	119.76 (14)
C5A—C4A—N17A	119.13 (12)	C10B—C9B—H9B	120.1
C6A—C5A—C4A	118.65 (13)	C8B—C9B—H9B	120.1
C6A—C5A—H5A	120.7	C11B—C10B—C9B	120.33 (14)
C4A—C5A—H5A	120.7	C11B—C10B—H10B	119.8
C5A—C6A—C1A	121.35 (13)	C9B—C10B—H10B	119.8
C5A—C6A—H6A	119.3	C10B—C11B—C12B	119.85 (14)
C1A—C6A—H6A	119.3	C10B—C11B—H11B	120.1
C1A—S7A—C8A	102.32 (6)	C12B—C11B—H11B	120.1
C9A—C8A—C13A	120.39 (13)	C11B—C12B—C13B	120.19 (14)
C9A—C8A—S7A	118.87 (11)	C11B—C12B—H12B	119.9
C13A—C8A—S7A	120.69 (11)	C13B—C12B—H12B	119.9
C8A—C9A—C10A	119.57 (13)	C12B—C13B—C8B	119.66 (14)
C8A—C9A—H9A	120.2	C12B—C13B—H13B	120.2
C10A—C9A—H9A	120.2	C8B—C13B—H13B	120.2
C11A—C10A—C9A	119.95 (14)	O16B—C14B—O15B^i^	126.33 (12)
C11A—C10A—H10A	120.0	O16B—C14B—C2B	116.29 (12)
C9A—C10A—H10A	120.0	O15B^i^—C14B—C2B	117.37 (12)
C12A—C11A—C10A	120.55 (14)	C14B^i^—O15B—Cu1	121.32 (9)
C12A—C11A—H11A	119.7	C14B—O16B—Cu1	123.77 (9)
C10A—C11A—H11A	119.7	O18B—N17B—O19B	123.72 (12)
C11A—C12A—C13A	119.84 (14)	O18B—N17B—C4B	118.56 (12)
C11A—C12A—H12A	120.1	O19B—N17B—C4B	117.72 (12)
C13A—C12A—H12A	120.1		
			
C6A—C1A—C2A—C3A	2.85 (19)	C6B—C1B—C2B—C3B	2.74 (19)
S7A—C1A—C2A—C3A	−178.32 (10)	S7B—C1B—C2B—C3B	−175.48 (10)
C6A—C1A—C2A—C14A	−177.17 (12)	C6B—C1B—C2B—C14B	−178.72 (12)
S7A—C1A—C2A—C14A	1.66 (18)	S7B—C1B—C2B—C14B	3.05 (18)
C1A—C2A—C3A—C4A	0.4 (2)	C1B—C2B—C3B—C4B	−0.8 (2)
C14A—C2A—C3A—C4A	−179.61 (12)	C14B—C2B—C3B—C4B	−179.46 (12)
C2A—C3A—C4A—C5A	−3.2 (2)	C2B—C3B—C4B—C5B	−1.8 (2)
C2A—C3A—C4A—N17A	178.95 (12)	C2B—C3B—C4B—N17B	177.03 (12)
C3A—C4A—C5A—C6A	2.6 (2)	C3B—C4B—C5B—C6B	2.4 (2)
N17A—C4A—C5A—C6A	−179.52 (12)	N17B—C4B—C5B—C6B	−176.42 (12)
C4A—C5A—C6A—C1A	0.8 (2)	C4B—C5B—C6B—C1B	−0.4 (2)
C2A—C1A—C6A—C5A	−3.4 (2)	C2B—C1B—C6B—C5B	−2.1 (2)
S7A—C1A—C6A—C5A	177.77 (11)	S7B—C1B—C6B—C5B	176.08 (11)
C6A—C1A—S7A—C8A	7.14 (13)	C6B—C1B—S7B—C8B	−5.99 (13)
C2A—C1A—S7A—C8A	−171.63 (11)	C2B—C1B—S7B—C8B	172.19 (11)
C1A—S7A—C8A—C9A	108.76 (12)	C1B—S7B—C8B—C13B	−89.83 (12)
C1A—S7A—C8A—C13A	−73.62 (12)	C1B—S7B—C8B—C9B	92.16 (12)
C13A—C8A—C9A—C10A	0.5 (2)	C13B—C8B—C9B—C10B	3.4 (2)
S7A—C8A—C9A—C10A	178.11 (11)	S7B—C8B—C9B—C10B	−178.58 (12)
C8A—C9A—C10A—C11A	1.5 (2)	C8B—C9B—C10B—C11B	−0.5 (2)
C9A—C10A—C11A—C12A	−1.5 (2)	C9B—C10B—C11B—C12B	−2.1 (2)
C10A—C11A—C12A—C13A	−0.6 (2)	C10B—C11B—C12B—C13B	1.7 (2)
C11A—C12A—C13A—C8A	2.6 (2)	C11B—C12B—C13B—C8B	1.2 (2)
C9A—C8A—C13A—C12A	−2.6 (2)	C9B—C8B—C13B—C12B	−3.7 (2)
S7A—C8A—C13A—C12A	179.85 (11)	S7B—C8B—C13B—C12B	178.26 (11)
C3A—C2A—C14A—O15A^i^	138.82 (13)	C3B—C2B—C14B—O16B	−162.85 (12)
C1A—C2A—C14A—O15A^i^	−41.16 (18)	C1B—C2B—C14B—O16B	18.58 (19)
C3A—C2A—C14A—O16A	−39.11 (17)	C3B—C2B—C14B—O15B^i^	17.83 (18)
C1A—C2A—C14A—O16A	140.91 (13)	C1B—C2B—C14B—O15B^i^	−160.74 (13)
O15A^i^—C14A—O16A—Cu1	−5.3 (2)	O15B^i^—C14B—O16B—Cu1	6.7 (2)
C2A—C14A—O16A—Cu1	172.44 (8)	C2B—C14B—O16B—Cu1	−172.57 (9)
C3A—C4A—N17A—O18A	−179.13 (13)	C3B—C4B—N17B—O18B	172.27 (13)
C5A—C4A—N17A—O18A	2.94 (19)	C5B—C4B—N17B—O18B	−8.87 (19)
C3A—C4A—N17A—O19A	0.73 (19)	C3B—C4B—N17B—O19B	−8.25 (19)
C5A—C4A—N17A—O19A	−177.20 (13)	C5B—C4B—N17B—O19B	170.61 (13)

Symmetry code: (i) −*x*+1, −*y*+2, −*z*.

### Hydrogen-bond geometry (Å, º)

*Cg*4 is the centroid of the C8*B*–C13*B* ring.

**Table:**

*D*—H···*A*	*D*—H	H···*A*	*D*···*A*	*D*—H···*A*
C6*B*—H6*B*···O18*A*^ii^	0.95	2.65	3.2785 (17)	124
C9*B*—H9*B*···O19*B*^iii^	0.95	2.39	3.2351 (19)	148
C10—H10*A*···*Cg*4^iv^	0.95	2.63	3.4171 (17)	140

Symmetry codes: (ii) −*x*+1, −*y*+1, −*z*+1; (iii) −*x*+1, −*y*+2, −*z*+1; (iv) −*x*+1, −*y*+1, −*z*.
